# Photosynthesis – beyond the leaf

**DOI:** 10.1111/nph.18671

**Published:** 2023-01-06

**Authors:** Tracy Lawson, Alexandra L. Milliken

**Affiliations:** ^1^ School of Life Sciences University of Essex Colchester CO4 3SQ UK

**Keywords:** net CO_2_ assimilation rate (*A*), nonfoliar photosynthesis, stomatal conductance (*g*_s_), stomatal density, wheat ears

## Abstract

Although leaves are considered the main site for photosynthesis, other green nonfoliar tissues can carry out considerable amounts of photosynthetic carbon assimilation. With photosynthesis, a potential target for improving crop productivity, physiology and contribution of nonfoliar tissues to overall plant carbon acquisition is gaining increasing attention. This review will provide an overview of nonfoliar photosynthesis, the role of stomata in these tissues and methodologies for quantification and the contribution to overall carbon gain.

**Table:** 

	Contents	
	Summary	55
I.	Introduction	55
II.	Methods for assessing nonfoliar photosynthesis	56
III.	Role of stomata in nonfoliar tissue	58
IV.	Conclusion	60
	Acknowledgements	60
	References	60

## I. Introduction

Photosynthesis has been studied in numerous species and environments, and the key components and pathways are well established. However, to date, the majority of these studies have focused on leaves, and few have considered the contribution from other tissues (Simkin *et al*., 2020). Green tissues other than leaves, including stems (Simkin *et al*., 2020), ears/panicles (Maydup *et al*., 2014; Rivera‐Amado *et al*., 2020; Zhang *et al*., 2022), green floral organs (Bertolino *et al*., 2022), pods (Wang *et al*., 2016) and fruit (Simkin *et al*., 2020), have been reported to photosynthesize and contribute to varying degrees and to overall plant carbon gain. Wheat and rice panicles, which a large proportion of studies have focused on, have been reported to have high photosynthetic capacities (Brazel & Ó'Maoiléidigh, 2019; Chang *et al*., 2020; Sanchez‐Bragado *et al*., 2020), with panicle and ear photosynthesis reported to contribute between 10% and 60% to yield (Hu *et al*., 2019) and spike photosynthesis positively correlated with yield (Molero & Reynolds, 2020). Rates of CO_2_ assimilation in nonfoliar organs depend on the material, conditions and methodologies used. A recent review by Araus *et al*. (2021) collated data on a number of C_3_ crop species (including rice, barley and wheat) and reported values between 0.6 and 30.3 μmol m^−2^ s^−1^ depending on the tissue and conditions, representing between 10% and 600% of flag leaf rates. Although typically rates of ear photosynthesis per unit area are often lower than in the flag leaf, these organs have a relatively large surface area, and when the total area is taken into account, these rates can be considerably higher (Araus *et al*., 2021). Considerable differences in mass between leaves or sources vs ears/pods and sink tissue also exist, for example the dry weight of a pea pod can be *c*. 10× greater than leaves.

In the last decade, manipulation of key processes to increase photosynthesis in leaves has been a prime objective to improve crop yields. Extending this to photosynthesis in ears could provide a novel target, and this potential has been demonstrated by Simkin *et al*. (2020) in wheat plants overexpressing the Calvin cycle enzyme SBPase, who reported ear photosynthesis, as well as leaf photosynthesis, was increased (Driever *et al*., 2017). Furthermore, natural variation in leaf photosynthetic capacity is known to exist between species (Weyers & Lawson, 1997) and cultivars (Faralli *et al*., 2019a,b) and similar variation has also been reported for ears and panicles of C_3_ crops (Molero & Reynolds, 2020; Tambussi *et al*., 2021) providing exciting opportunities to exploit such natural variation for on‐going breeding programs.

The importance and contribution of ear or spike photosynthesis (and other potential nonfoliar green tissue) to yield is considered even more important when foliar tissues (and particularly the flag leaf, which is considered the major photosynthetic contributor to grain filling) are damaged or stressed (Ntakirutimana & Xie, 2020). For example, Zhang *et al*. (2011) reported that nonfoliar organs in wheat accounted for 27–62% of the total green area per culm and this ratio increased significantly with reduced water availability. Under water‐stressed conditions, wheat flag leaf photosynthetic capacity and rate decrease along with key photosynthetic enzymes; however, the same is not true for ears or awns (Vicente *et al*., 2018), with ear photosynthesis being maintained (Tambussi *et al*., 2005) and critical to grain filling and yield maintenance under such environmental conditions (Hu *et al*., 2019). A possible explanation for the differential impacts of water stress on ears compared with leaves is that water relations in these organs are considerably different to foliar tissue and stomata could play a key role in this regulation (to be described later).

**Fig. 1:**
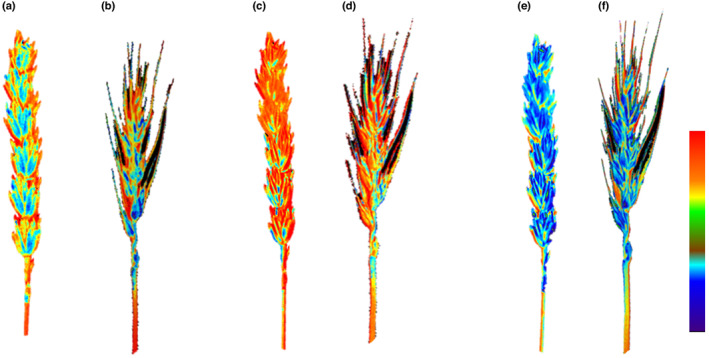
Chlorophyll fluorescence images of awned and nonawned wheat ears of (a, b) photosystem II (PSII) operating efficiency ($F_{q}^{\prime }$/$F_{m}^{\prime }$); (c, d) $F_{q}^{\prime }$/$F_{v}^{\prime }$ (PSII photochemical quenching factor); and (e, f) maximum PSII operating efficiency ($F_{v}^{\prime }$/$F_{m}^{\prime }$). Colour scale bar represents 0.4–0.6 for $F_{q}^{\prime }$/$F_{m}^{\prime }$ and 0.5–0.85 for $F_{q}^{\prime }$/$F_{v}^{\prime }$ and $F_{v}^{\prime }$/$F_{m}^{\prime }$.

Wheat ears are made up of a number of photosynthetic components, and significant variation in assimilation rates exists between these different structures. Fig. 1 shows chlorophyll fluorescence (CF) measurements of photosynthetic processes and demonstrates differences in efficiency between awns and the other wheat ear components. The glumes appear to have the highest photosynthetic efficiency (Fig. 1a) compared with the awns (Fig. 1b), although both are considered important in terms of photosynthetic contribution (see Hu *et al*., 2019). Spatial differences in photosynthetic efficiency are due mostly to photochemical quenching (Fig. 1c,d) rather than differences in $F_{v}^{\prime }$/$F_{m}^{\prime }$ which decreases with nonphotochemical quenching (Fig. 1e,f), suggesting that carbon fixation is an important sink for the end products of electron transport. Photosynthetic contribution from awns is thought to be particularly important in stress conditions, and therefore, awned varieties may be advantageous in environments that experience periodic stresses (Ntakirutimana & Xie, 2020). It is also well established that genotypic variation in ear water‐stress tolerance exists (Li *et al*., 2017), which could represent another currently unexploited target for breeding programs to develop wheat ideotypes for future climatic conditions.

## II. Methods for assessing nonfoliar photosynthesis

One of the major restrictions in quantifying the contribution of nonfoliar photosynthesis to overall carbon assimilation and yield is methodological limitation. This is further complicated by different photosynthetic pathways operating in these tissues. Here, we outline some of the complications and options for measuring photosynthetic carbon assimilation in organs and provide some insights into possible new approaches.

### 1. Complexities of measurements

**Fig. 2:**
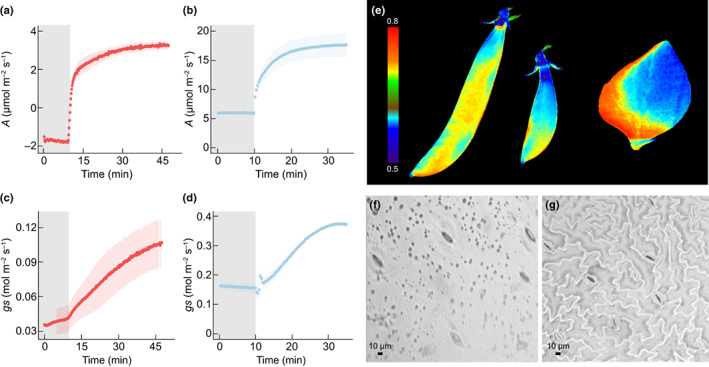
Example of photosynthetic activity in Cameor pea pods and leaves. Mean (a) pod and (b) leaf assimilation (*A*), (c) pod and (d) leaf stomatal conductance (*g*_s_) was measured in response to a step in light intensity from 100 to 1000 μmol m^−2^ s^−1^ photosynthetic photon flux density (PPFD), at 400 ppm CO_2_ and 23°C within a bespoke pod chamber. Grey shaded areas represent when the light source is at 100 μmol m^−2^ s^−1^ PPFD. Error bars represent mean ± SE (*n* = 3). All measurements are normalized to an illuminated projected area (typically used for leaves). (e) Chlorophyll fluorescence image of photosystem II operating efficiency ($F_{q}^{\prime }$/$F_{m}^{\prime }$) was used to demonstrate differences in efficiency between the two tissue types. Colour scale bar represents an $F_{q}^{\prime }$/$F_{m}^{\prime }$ of 0.5–0.8. Example of a (f) pod and (g) leaf epidermal impression at a 200× magnification.

Typically, leaf measurements of photosynthesis (*A*) and transpiration are made using infra‐red gas analysers, which enclose a flat leaf or proportion of a leaf into a cuvette and use differential measures of gases entering and leaving the chamber. The development of bespoke chambers that enable gas exchange measurements in complex organs (wheat ears and rice panicles) is providing new information on photosynthetic capacity, stomatal kinetics and WUE (Maydup *et al*., 2010; Chang *et al*., 2020; Henry *et al*., 2020). An example of these types of measurements is illustrated in Fig. 2, which shows dynamic responses of *A* and *g*_s_ in pea pods following a step change in light intensity (Fig. 2a,b). This figure also illustrates that even with 100 μmol m^−2^ s^−1^ PPFD, pod photosynthesis is negative and therefore well below the light compensation point, indicative of high rates of respiration. Infra‐red gas analyser measurements of nonfoliar material are not without complications. First, leaves are usually a flat lamina surface and easily sealed into a chamber, whilst fruits, pods and stems vary greatly in size, shape and dimension. A second problem is that photosynthesis is measured on a projected leaf area basis, and the 3D structure of ears and fruits can make quantification of the area challenging. Furthermore, the complexity in shape of the material can result in uneven illumination (Hu *et al*., 2019; Chang *et al*., 2020), and temperatures, further complicating measurements. 3D scanning can be used to overcome the problem of establishing leaf area (Simkin *et al*., 2020), whilst surrounding chamber LED illumination reduces shading. Gas exchange measurements also require knowledge of boundary lay conductance, which is difficult to determine for nonlamina material, but can be measured using the filter paper methods (Parkinson, 1985) and mimicking the shape of the organ.

A considerably more difficult problem to overcome when measuring nonleaf photosynthesis is the controversy around the mechanisms of photosynthesis. Although the majority of studies have assumed a similar pathway to C_3_ leaf mesophyll photosynthesis, this may not always be the case. In nonfoliar tissue, it has been proposed that there are two major sources of CO_2_ for photosynthesis. Atmospheric CO_2_ can diffuse from the atmosphere into the cells through stomatal pores and fixed by Rubisco, as per C_3_ foliar photosynthesis. However, a second supply of CO_2_ for fixation is also released from high mitochondrial respiration and subsequently refixed (Millar *et al*., 2011), which would underestimate photosynthetic rates measured by gas exchange. The extent to which each of these pathways contributes to photosynthesis is under debate and most likely species‐specific. Refixation of respiratory CO_2_ may be particularly important in internal tissues where atmospheric C‐fixation is restricted (i.e. the seeds), due to longer CO_2_ diffusion pathways and reduced light penetration (Henry *et al*., 2020; Simkin *et al*., 2020). Higher rates of respiration in panicles compared with leaves (Chang *et al*., 2020) suggest a substantial contribution of respiratory CO_2_ to photosynthesis (Sanchez‐Bragado *et al*., 2020; Zhang *et al*., 2022) in these tissues, with reports of between 55% and 75% of respired CO_2_ refixed in wheat and barley (Bort *et al*., 1996).

### 2. Indirect methods for assessing nonfoliar photosynthesis

There are several indirect methods for assessing the contribution of ear and stem photosynthesis, including organ removal and shading, where the ear or stems are covered with foil to reduce light penetration (Maydup *et al*., 2010). However, shading alters temperature and gaseous flow, whilst removing organs can induce stress responses, both of which impact on photosynthetic rates (Tambussi *et al*., 2021). Quantification of O_2_ accumulation as an indication of electron transport has been employed to assess different ear components; however, this requires destructive sampling rather than *in situ* measurements (review by Tambussi *et al*. (2021)). Chlorophyll fluorescence imaging is another tool that can be utilized to measure photosynthetic efficiency (and calculate electron transport rate) in both leaf and nonleaf material (Tambussi *et al*., 2005; Simkin *et al*., 2020; Figs 1, 2). Although this is a rapid and relatively simple approach, electron transport is only a proxy for carbon assimilation and alternative electron sinks including photorespiration can influence photosynthetic efficiency (Murchie & Lawson, 2014). It may therefore be advantageous to combine CF measurements with gas exchange to determine the proportion of electrons used to fix atmospheric CO_2_ from those going to alternative sources. This can be easily achieved by performing measurements under 2% O_2_ to eliminate photorespiration (McAusland *et al*., 2013, 2015, 2019), although the amount of CO_2_ produced through respiratory processes may complicate this. Using membrane inlet mass spectrometry could provide an advanced approach to quantifying different processes. Utilises naturally occurring stable isotopes of O_2_ and CO_2_ and using enrichment approaches would enable discrimination from respiration from other O_2_ consumption, as well as O_2_ evolution from photosynthetic electron transport under different conditions (Driever & Baker, 2011). Thermal imaging can provide insights into spatial and temporal stomatal behaviour, that may be valuable to elucidate and quantify atmospheric CO_2_ fixation from other CO_2_ sources. Combined thermography with other imaging approaches such as CF (McAusland *et al*., 2013, 2015) could deliver further insights into water‐use efficiency in different tissues (McAusland *et al*., 2013). For a recent review on different methods for measuring ear photosynthesis, we refer readers to Tambussi *et al*. (2021).

## III. Role of stomata in nonfoliar tissue

**Fig. 3:**
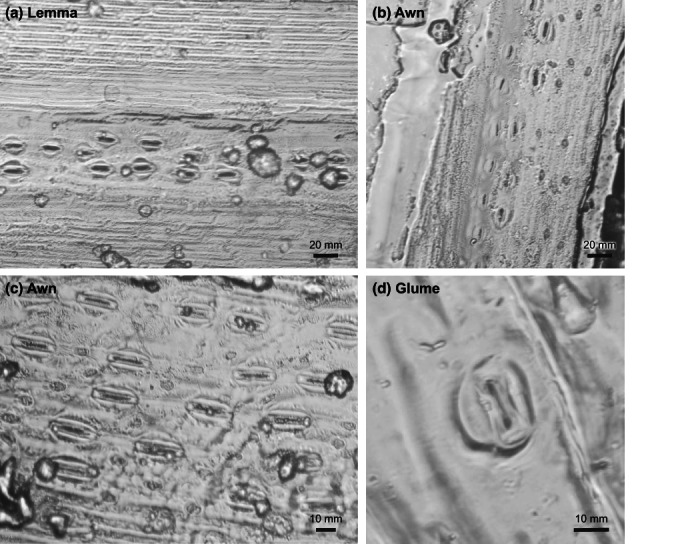
Example of epidermal impressions showing stomata from a barley (a) lemma and (b) awn and a wheat (c) awn and (d) glume.

In leaves, photosynthesis requires atmospheric CO_2_ to enter the leaves through the stomatal pores and subsequently stomatal density (SD) and behaviour influence assimilation rate and stomatal conductance, which is closely correlated with rates of carbon fixation. For some nonleaf tissues, such as tomato fruit, the lack of stomata highlights the sole reliance upon carbon refixation (Simkin *et al*., 2020). Stomata are found in various numbers on nonfoliar tissues, such as rice panicles (Li *et al*., 2021; Rangan *et al*., 2022), wheat stems and ear components (Fig. 3; Hu *et al*., 2019; Henry *et al*., 2020; Simkin *et al*., 2020), and certain fruits (Brazel & Ó'Maoiléidigh, 2019); however, their functionality, including their contribution to carbon acquisition, has not been fully evaluated (Simkin *et al*., 2020). Recent work by Bertolino *et al*. (2022) has reported considerable spatial variation in stomata in rice floral organs and suggested that they are morphologically distinct from leaf stomata, although their function is still unknown.

**Fig. 4:**
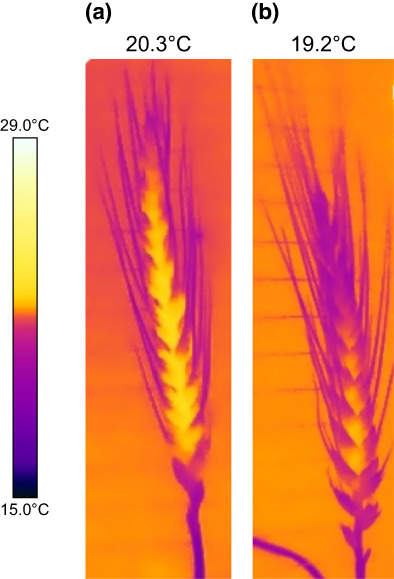
Thermal images of wheat ears which demonstrate functional difference due to variation in stomatal density (SD): (a) SD of 12 mm^−2^ and (b) 35 mm^−2^. Measurements were made following 1 h exposure to 27°C. Colour scale bar represents a difference in temperature from 15°C to 29°C.

Using thermography Simkin *et al*. (2020) showed that stomata of wheat ears were functional and responded to changes in light intensity; similar to leaves, albeit *g*_s_ levels were lower. The existing variation in SD on different tissue types may allude to functional differences. For example, in Fig. 4, we show that wheat ears with a lower SD have higher ear temperatures than those with greater stomatal numbers (Fig. 4). Fruits have been reported to have 1–10% of the density found in leaves, with apples even lower numbers at 30 times less than leaves (Blanke & Lenz, 1989). However, SD in wheat ear organs is only 50–60% lower than that in the leaf (Tambussi *et al*., 2005), and some studies have even reported higher SD in ears than leaves. Interestingly, stomata have been found on both the adaxial and abaxial sides of glumes, lemma (Simkin *et al*., 2020) and bracts (Ding *et al*., 2018). Ding *et al*. (2018) proposed that adaxial bract stomata facilitate CO_2_ uptake from the respiring grain. Such amphistomaty could indicate stomata function to support both atmospheric CO_2_ uptake from one side of the tissues and refixation of respiratory CO_2_ uptake on the other. Such functionality could be key to maintaining ear photosynthesis under stressful conditions that cannot be achieved by the flag leaf. This hypothesis is supported by a recent study by Zhang *et al*. (2022) who explored control of photosynthesis in rice panicles at different stages of crop development and reported that at anthesis, panicle photosynthesis was dependent on both *g*_s_ and biochemical function, and there was a positive correlation between *g*_s_ and *A*. However, at grain filling, *g*_s_ declined and net photosynthesis was correlated with *g*_s_ rather than the carboxylation capacity of Rubisco (*Vc*_max_), indicating that *A* was primarily determined by stomatal behaviour. Respiration in panicles has been reported to increase in the initial stages of grain filling along with a decrease in *g*_s_, supporting the idea that respiratory CO_2_ is important for panicle photosynthesis at this stage (Chang *et al*., 2020). This suggests that there is a possible switch between atmospheric CO_2_ being the main supply for photosynthesis earlier in the season supported by higher *g*_s_, which switches to respiratory supply later in development, concurrent with a decrease in *g*_s_. At the same time, biochemical changes support refixation of respired CO_2_ (Zhang *et al*., 2022). However, changes in panicle *g*_s_ could also be due to shifts in the osmotic and water status of the panicle. Xylem water potential would be decreased with phloem unloading in the panicles at grain filling and could explain a decrease in *g*_s_.

Although, to date, the majority of research on stomata in nonfoliar photosynthesis has focused on their role in CO_2_ uptake, stomata are also important for transpiration and the movement of water through the plants, including the translocation of photoassimilates (Simkin *et al*., 2020). Stomatal control of evaporative cooling in all plant tissues is critical for maintaining temperature for photosynthesis and reproductive capacity. It is well established that heat stress greatly impacts wheat yield, with flower and reproductive growth phases being particularly sensitive (Yadav *et al*., 2022). Heat stress, several days before anthesis during floral development, greatly affects ovule and pollen formation with significant impacts on grain development and yield. It has been proposed that ear temperature during the early stages of anthesis is an important component of heat stress tolerance (Steinmeyer *et al*., 2013), and therefore, stomatal behaviour possibly could be critical for evaporative cooling and maintenance of temperature at critical stages. Transpirational water loss through the stomata may also play a key role in the translocation of photoassimilates from sources to the ear sinks during grain filling, and the changes in osmotic/water potential during this process could be responsible for changes in *g*_s_ at different developmental stages. Furthermore, the amount of water lost through nonfoliar organs, although difficult to establish from current literature, needs to be quantified in order to fully appreciate overall crop water use. Further research is necessary to fully establish the role of stomata in these tissues, along with hydraulic capacity, and overall water loss and carbon gain in these organs.

Natural variation in SD between cultivars in leaves (Weyers & Lawson, 1997), ears (Li *et al*., 2017) and individual ear components (Simkin *et al*., 2020) provides an opportunity to explore the role of stomata further. Li *et al*. (2017) demonstrated that wheat cultivars with lower ear SD had increased WUE and drought tolerance. Therefore, manipulating SD (e.g. Bertolino *et al*., 2022) or function (Lawson & Matthews, 2020) in wheat ears alone could improve heat tolerance and support greater photosynthesis through increased evaporative cooling, influence translocation of photoassimilates and nutrients to the grains and provide a route to improve nonfoliar water‐use efficiency.

## IV. Conclusion

Although the contribution of nonfoliar tissues to carbon assimilation and yield has not been fully quantified and depends on tissue, cultivars and environmental conditions, it represents an exciting and (to date) mostly unexploited area for improved crop productivity. Furthermore, understanding and manipulating stomatal behaviour in these organs could provide a unique opportunity to produce crop cultivars with greater stress tolerances, through increased cooling capacity, greater translocation, higher photosynthesis and the ability to yield in environments that might otherwise be subject to significant losses; however, these could come at the expense of water‐use efficiency.

## Competing interests

None declared.
